# Ab initio studies of magnetism and topology in solid Pd-rich ${\varvec{a}}$-PdSi alloys

**DOI:** 10.1038/s41598-022-08656-5

**Published:** 2022-03-17

**Authors:** Isaías Rodríguez, Renela M. Valladares, Alexander Valladares, David Hinojosa-Romero, Ariel A. Valladares

**Affiliations:** 1 Instituto de Investigaciones en Materiales, Universidad Nacional Autónoma de México, Apartado Postal 70-360, 04510 Ciudad Universitaria, CDMX, Mexico; 2 Facultad de Ciencias, Universidad Nacional Autónoma de México, Apartado Postal 70-542, 04510 Ciudad Universitaria, CDMX, Mexico

**Keywords:** Magnetic properties and materials, Structure of solids and liquids, Theoretical physics, Density functional theory, Molecular dynamics

## Abstract

In 1965 Duwez et al. reported having generated an amorphous, stable phase of palladium-silicon in the region 15 to 23 atomic percent, at.%, silicon. These pioneering efforts have led to the development of solid materials that are now known as Bulk Metallic Glasses, BMG. In 2019 Rodríguez et al. discovered, computationally, that bulk amorphous Pd becomes magnetic, and so does porous/amorphous Pd. Puzzled by these results, the study of several solid binary systems in the Pd-rich zone was undertaken; in particular, the study of the glassy metallic alloy *a*-Pd$_{100-c}$Si$_{c}$, for $0 \le c \le 22$, (*c* in at.%) to see what their topology is, what their electronic properties are and to inquire about their magnetism. In this work it is shown that this metallic glass is in fact magnetic in the region $0 \le c < 15$. Collaterally $\alpha$ and $\beta$ magnetization curves are shown where the net magnetic moment is presented. The topology and the position of the first few peaks of the pair distribution functions, which agrees well with experiment, are also discussed. The BMGs produced experimentally so far are limited in size, but despite this limitation, recent industrial efforts have developed some useful devices that may revolutionize technology.

## Introduction

Ever since Klement, Jr. et al. generated an amorphous, unstable, phase of a gold-silicon alloy in 1960^1^, Au$_{75}$Si$_{25}$ in atomic percent, at.%, much has been written and even more has been done in the field of glassy metals. In September 3, 1960, Pol Duwez and his two graduate students W. Klement, Jr., and R. H. Willens reported that by rapid solidification of the liquid, a non-crystalline structure of AuSi could be generated. Thereafter, in 1965, Duwez et al.^2^ obtained stable, amorphous metallic alloys of palladium and silicon, *a*-Pd$_{100-c}$Si$_{c}$ for concentrations $15< c < 23$, using the same experimental approach as before; i.e., a rapid cooling from the melt. These are the beginnings of the production of glassy metals by rapid cooling from the liquid and the evolution of the field of Bulk Metallic Glasses, BMGs, was under way. The Pd-Si system is a prototypical, simple, example of this field.

In Duwez words, “in September 1959, ... as part of a research program whose purpose was far remote from metallic glasses, an alloy containing 75 at.% Au and 25 at.% Si rapidly quenched from the liquid state appeared to be amorphous.”^3^. A curiosity at first, with time it has become clear that glassy metals in general, and BMG in particular, have fascinating and potentially very useful properties^4^. For example, some of the spectacular properties deal with their resistance to wear, that allows the use of them in lasting gears with several applications, like in the food industry where the use of lubrication may lead to the contamination of the products. In collaboration with NASA, industries are working to develop gears from BMG for space modules subject to extreme weather conditions that restrict the use of common lubricants^5,6^. Their mechanical properties are also worth mentioning since they are very resistant to stresses^4^ and therefore more durable. So far the limiting factor is the small size of the BMGs produced, since the largest specimen generated is a BMG of Pd$_{42.5}$Cu$_{30}$Ni$_{7.5}$P$_{20}$ with dimensions no larger than 10 cm along any of the three spatial directions^7^.

Glass has been known for millennia^8^, but it was during the last century that metallic glasses began to appear and to claim their place in the scientific and technological scenario. However, since the science of glass is even more recent, it should not surprise anyone the limited knowledge of some of the properties of these materials; it is now that an understanding is beginning to appear as to why metallic glasses behave the way they do and the potential usefulness of amorphous solids in general. In the PdSi system, the oldest stable glassy binary prepared from the melt, Si is known as the glass forming element and ever since the amorphicity of this system was reported, studies have been conducted to understand their behavior. The PdSi alloys have been largely studied but there are features not well understood and some others to be researched.

The range of concentration considered by Duwez and coworkers, although very restricted, is illustrative enough to detonate the growth of diverse studies related to its properties and structure. The phase diagram for the palladium-silicon alloys indicates the presence of a eutectic structure at about 15 at.% Si and at a temperature of the order of 1090 K, and the amorphous alloys obtained range in concentrations from 15 to 23 at.% Si. It was Cohen and Turnbull that first pointed out that the proximity of the eutectic point was relevant to the formation of the amorphous structure^9,10^ and from there on people started to look for eutecticity in phase diagrams to generate new amorphous glassy metallic alloys. The relevance of this result is that for the first time, an amorphous material could be formed by very rapid cooling from the melt, unlike other processes known at the time, and it was found that for the 20 at.% Si alloy, undercooling as large as $300^{\circ }\hbox {C}$ could be reached^2^.

Despite obstacles, efforts continue to generate larger samples of BMGs to make them applicable in some everyday situations. When large samples of BMGs become available the technological possibilities will grow, and this is the quest in many laboratories around the world.

### Motives

**Figure 1:**
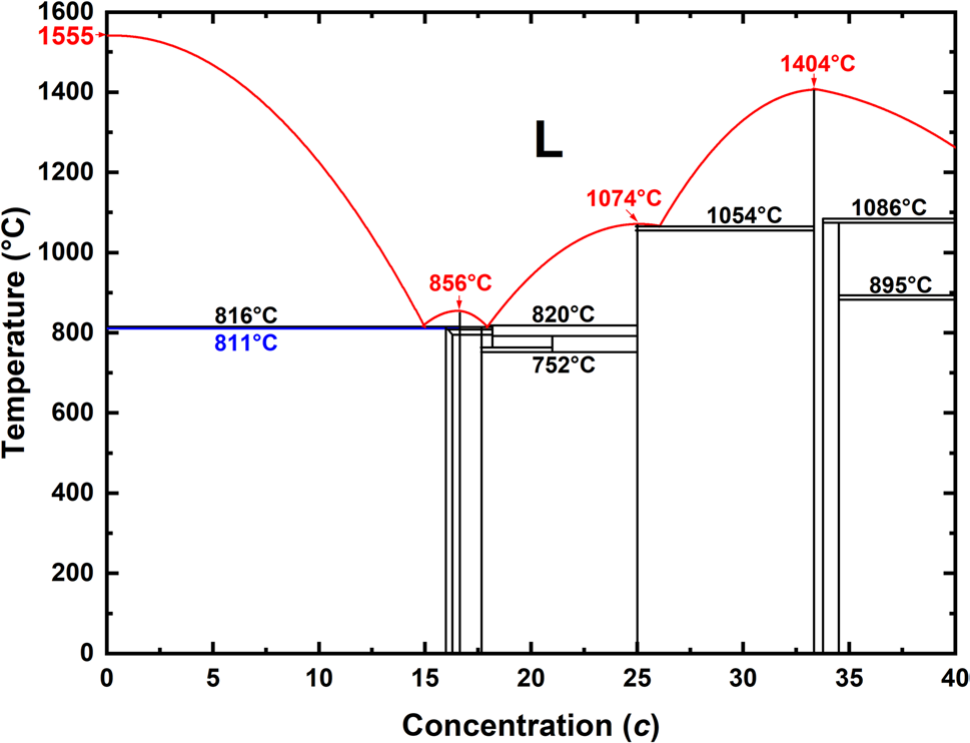
Region of the Pd-Si phase diagram near the eutectic point. The atomic concentrations deployed are $0 \le c \le 40$ at.%. This figure is a linear (black lines) and parabolic (red lines) fit to the experimental values contained in References^13–15^.

In 2019 Rodríguez et al. discovered that bulk amorphous Pd becomes magnetic^11^. Puzzled by our results some Pd-based amorphous materials were studied by us, for concentrations close to the palladium-rich zone to see if this magnetism would persist, and to what extent. Contaminating *a*-Pd with hydrogen, deuterium or tritium to generate palladium “ides”, *a*-Pd (H/D/T)$_{x}$, would be a natural path since Pd is well known for ad- and ab-sorbing hydrogen and its isotopes; to the point that it has been considered as an alternative to store H and use it in electrical vehicles. So in fact, contaminating with H, D, and T contributed to decrease the magnetism of amorphous Pd until, for values of the ratio *x* close to 50% the magnetism completely disappears, and superconductivity appears giving rise to the so-called inverse isotope effect for the three isotopes, H, D and T^12^. It was a fortunate circumstance that the Pd-rich zone for the *a*-PdSi is near a eutectic point that would explain, according to reference^9^, the appearance of amorphous structures; this region is depicted in Fig. 1, which is a linear (black lines) and parabolic (red lines) fit to the experimental values found in References^13–15^.

Magnetism in the liquid phase of palladium-silicon alloys has been studied by Müller et al. back in 1978^16^. They found that for all silicon concentrations magnetism appears and the magnetic susceptibility decreases with increasing silicon and becomes negative for concentrations larger than 20%. At $c \approx 60\%$ it becomes positive again and remains positive. They argue that due to the empirical similarity between glassy and liquid metals, magnetic inferences can be made concerning the solid glassy phases, and that therefore magnetism in the amorphous solid samples should appear in a similar manner and with a similar behavior as found for the liquid. However, no experimental, simulational or theoretical results have been found by the present authors, prior to our work. Sänger^17^ in 1984 analyzed the experimental results for the liquid and decomposed magnetism in para- and dia-, offering an explanation for the found, as will be shown later on.

The results of magnetism for the liquid alloys, plus the results found by our group for amorphous Pd, for porous/amorphous Pd, and for palladium hydrides, led us to the investigation of magnetism in these alloys. To the best of the authors’ knowledge, no work in the literature reports possible magnetic properties for the Pd-rich concentrations of the solid glassy palladium-silicon alloys. Also, in this work, their electronic properties and the densities of states for $\alpha$ (spin up) and $\beta$ (spin down) spins are reported. Since there seems to exist a discrepancy in the experimental positions of the maxima of the first few peaks in the Pair Distribution Functions, PDFs, and some simulated results^18,19^; these parameters are also analyzed. The work hereby described was conducted for *a*-Pd$_{100-c}$Si$_{c}$, with concentrations in the interval $0 \le c \le 22$.

## Methods

To generate the amorphous Pd-Si samples the *undermelt-quench* procedure was used, a molecular-dynamics approach developed in our group that has led to very good specimens of the amorphous phases^11,20^. Alloys with 8 different concentrations of silicon (*c* = 2.5, 5, 10, 13.34, 15, 17.5, 20 and 22) were randomly arranged in a (non-stable) diamond-like supercell containing a total of 216 atoms of both elements. Care was exercised to construct these supercells using the experimental densities reported in the literature^13,18,21^. Then using CASTEP^22^ included in the Materials Studio suite of codes^23^ Molecular Dynamics processes, MDs, were performed starting from the diamond-like non-equilibrium structures to propitiate the randomization of the structures. Once the MDs cycles were completed and the amorphization procedure finished, the atomic structures underwent Geometry Optimization, GO, searching for the topology that would locally minimize the energy. Clearly, the amorphous arrangement is not the *minimum-minimorum* of the energy; such minimum energy structure would be the crystalline one. In this manner, when the final atomic distributions were attained, the structures were *locally* stable and amorphous.

### Specifics

The code CASTEP^22^ of the Materials Studio suite of codes^23^ was utilized for MD and GO processes. The exchange-correlation treatment was done with the Perdew-Burke-Ernzerhof, PBE, form of the Generalized Gradient Approximations, GGA, functional^24^ and the ultrasoft pseudopotentials^25^ included in the Materials Studio suite were used in both MD and GO. A Pulay mixing scheme was employed both for the MD and GO with a thermal smearing of 0.1 eV for the occupation, together with a Self-Consistent Field, SCF, energy threshold of $2.0 \times 10^{-6}$ eV.

For the MD processes, the NVT ensemble with the Nosé-Hoover thermostat was used with a time step of 7 fs. The disordering thermal processes consist of a heating ramp of 100 steps, starting at 300 K for all samples and reaching 1000 K for $c > 10$ at.% (1500 K for $c \le 10$ at.%), a thermal slope of 7 K/step for $c > 10$ at.% (12 K/step for $c \le 10$ at.%), staying always below the liquidus temperature. After the heating ramps, cooling ramps (with the same (absolute value) slope as the heating) of 125 steps were performed from the highest temperature to 7 K for $c > 10$ at.% (12 K for $c \le 10$ at.%), for a total of 225 steps of thermal process. The total MD simulation time for a typical heating and cooling cycle was 1.575 ps. The volumes and densities considered are listed in Table 1. For the plane-wave basis a 300 eV cut-off energy, with a grid-scale of 2.0 and a fine-grid-scale of 3.0, was used; the grid scales determine the size of the standard and fine grid for the G-vectors, relative to the diameter of the cutoff sphere.

For the GO of the amorphous structures the following parameters were used: a plane-wave basis of 330 eV for the cut-off energy, with the same grid scales for the MD. The total spin of the specimens was not fixed during the heating and the cooling procedure, and neither during the GO processes, so the final structures were obtained with the spin unrestricted to allow for the evolution dictated by the interactions and the procedure.

The SCF energy threshold was $5.0 \times 10^{-7}$ eV and for the Broyden-Fletcher-Goldfarb-Shanno, BFGS, minimizer (using delocalized internals) the following tolerances were employed: energy tolerance of $5.0 \times 10^{-7}$ eV, force tolerance of $1.0 \times 10^{-2}$ eV Å$^{-1}$, and a maximum displacement of $5.0 \times 10^{-4}$ Å.

**Table 1:** Lattice parameters, volumes and densities of the supercells used for the various concentrations.

Alloy	Lattice parameter	Volume	Density
	$a=b=c$ (Å)	(Å$^3$)	(g cm$^{-3}$)
Pd$_{78}$Si$_{22}$	14.56	3086.12	10.31
Pd$_{80}$Si$_{20}$	14.57	3090.25	10.54
Pd$_{82.5}$Si$_{17.5}$	14.59	3107.73	10.69
Pd$_{85}$Si$_{15}$	14.64	3137.59	10.83
Pd$_{86.66}$Si$_{13.34}$	14.64	3138.30	10.96
Pd$_{90}$Si$_{10}$	14.70	3177.51	11.11
Pd$_{95}$Si$_{5}$	14.71	3181.17	11.51
Pd$_{97.5}$Si$_{2.5}$	14.72	3191.37	11.71
Pd$_{100}$	14.71	3180.28	12.00

### Calculations

At the end of the MD and GO processes the $\alpha$ spins and $\beta$ spins were determined to investigate the possible magnetism of these alloys. All results are reported in the next section. A collateral product of this investigation is the comparison of the positions of the first few prominent peaks of the PDFs with some results obtained in 1975^26^, 1980^27^, 1981^19^, 2003^28^ and in 2012^29^, as will be shown. At the time, there were discrepancies between the experimental values measured and those obtained in some simulations.

## Results

**Figure 2:**
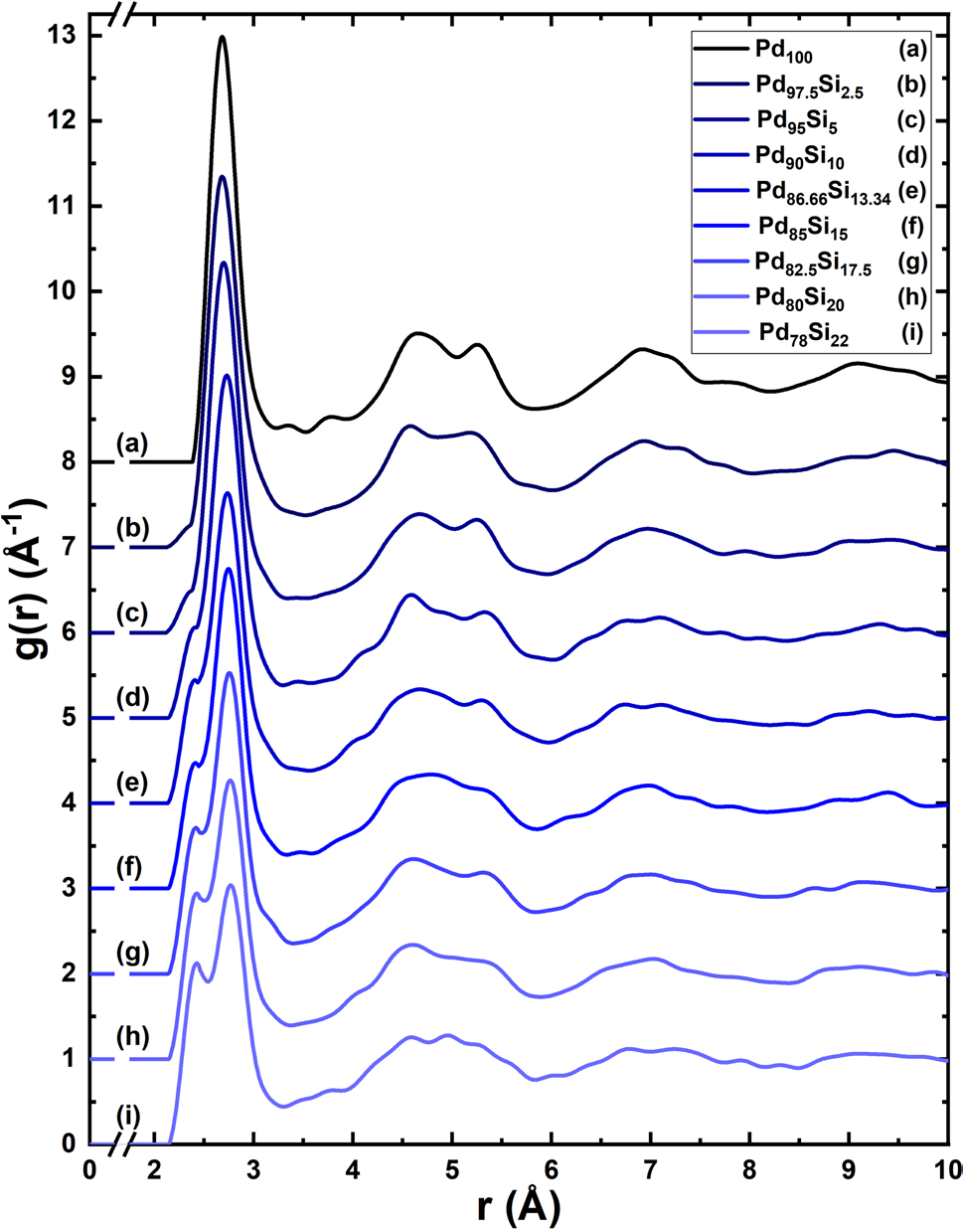
Total pair distribution functions, tPDFs, for the 8 alloys studied in this work and for the pure bulk palladium sample. The bimodal character of the second peak, typical of amorphous Pd, gradually disappears as the silicon concentration increases. The tPDFs were calculated using Correlation, an open-source software developed by Rodríguez et al.^31^.

**Figure 3:**
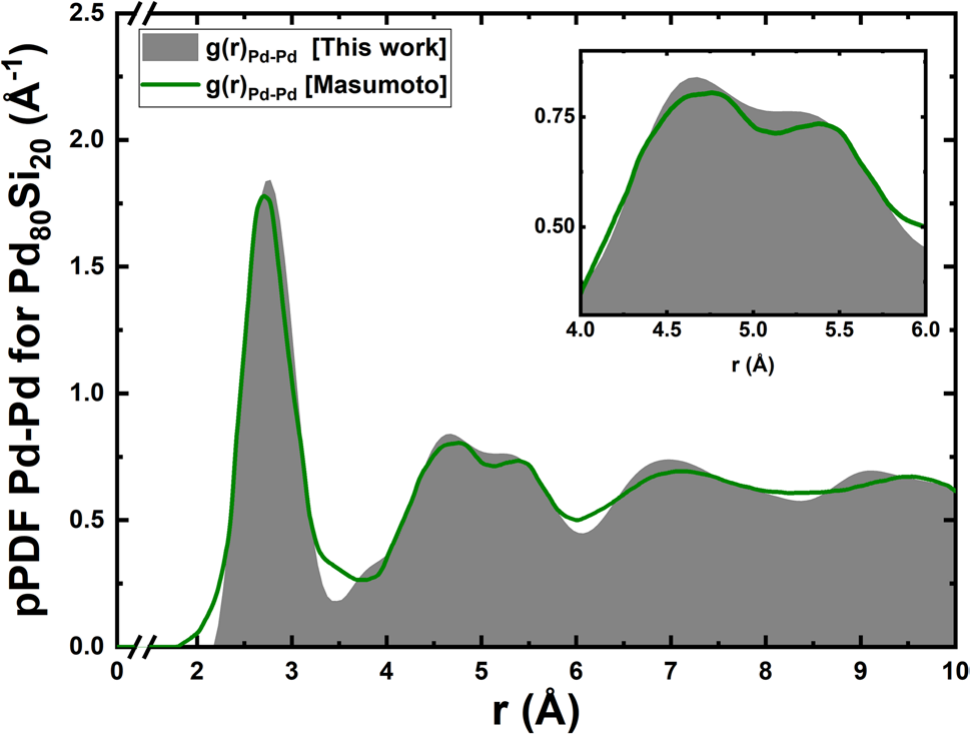
Comparison of the Pd-Pd pPDF for the Pd$_{80}$Si$_{20}$ sample with the partial experimental result of Masumoto (in Waseda’s book^27^)^26^. The bimodal elephant-like nature of the second peak is depicted in the inset.

Figure 2 represents the total Pair Distribution Functions, tPDFs, of the nine supercells studied in this work, 8 amorphous alloys plus the pure amorphous palladium sample^11^. The 216-atom initially unstable supercells have a diamond-like structure with densities determined by experiment^13,18,21^ and an edge length that goes from 14.55 Å for the Pd$_{78}$Si$_{22}$ to 14.71 Å for the Pd$_{100}$ sample. PDFs are difficult to obtain experimentally but are the best global description of the amorphous atomic topology of pure elements and alloys. In particular, the partial PDFs, pPDFs, require more labor by the experimentalists and they are not as frequently reported as the total. In our approach partial and total PDFs were obtained. In a previous paper by Alvarez et al.^30^ the assumptions that experimentalists have to invoke to describe partial PDFs were constrasted with our approach and demonstrated that this is far superior than to assume Gaussian curves fitted to the position of the peaks observed in the PDFs. The agreement of the silicon low concentration PDFs with the experimental results included in Ref.^11^ for pure amorphous Pd is to be noted, and indicates consistent results in our simulations, see Fig. 3.

**Figure 4:**
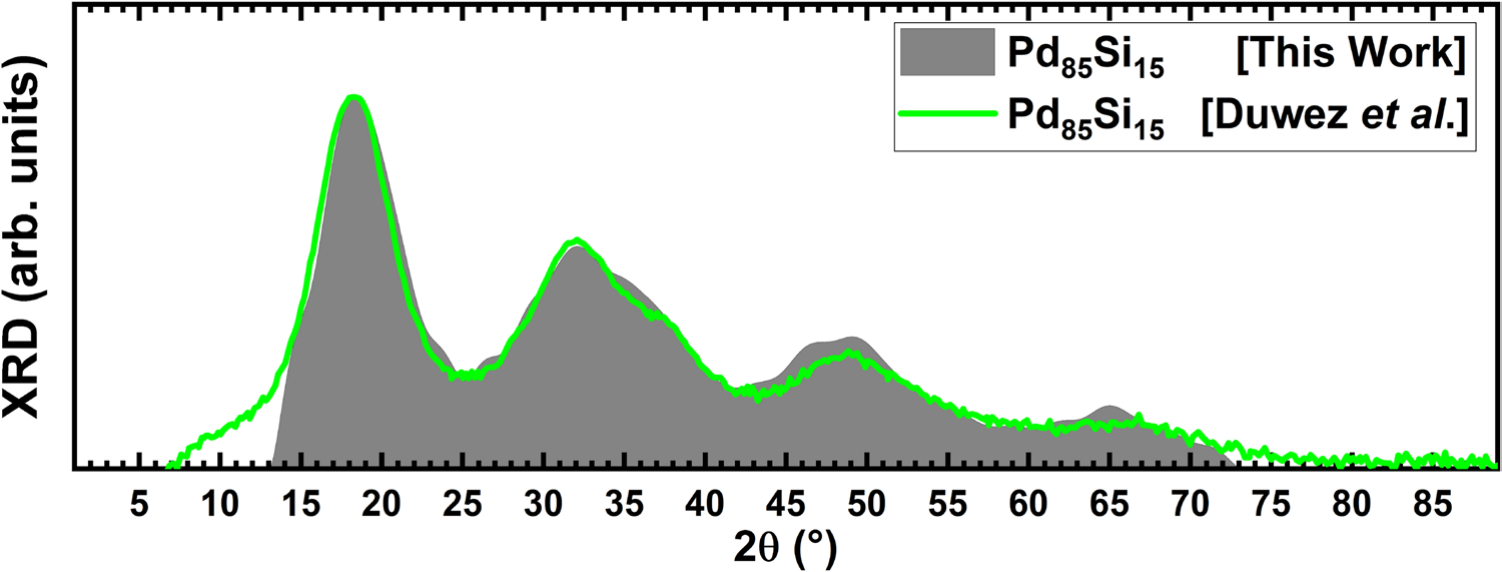
Comparison of the XRD pattern obtained from the experimental results of Ref.^2^ ($c = 15$ at.%), green line, with our simulations, dark solid profile. See text.

To compare with the results of Duwez et al.^2^, the X-Ray Diffraction pattern, XRD, was calculated using Reflex, a package included in the Materials Studio suite of codes. Since the units are arbitrary the first peak of our simulation was superimposed to the first peak of the experimental results and then both curves reasonably coincided for most of the angles considered in the experiment, Fig. 4. Since Duwez et al. did this XRD study for *a*-PdSi with a silicon concentration of 15% the comparison is done with one of our samples constructed for the same concentration.

**Figure 5:**
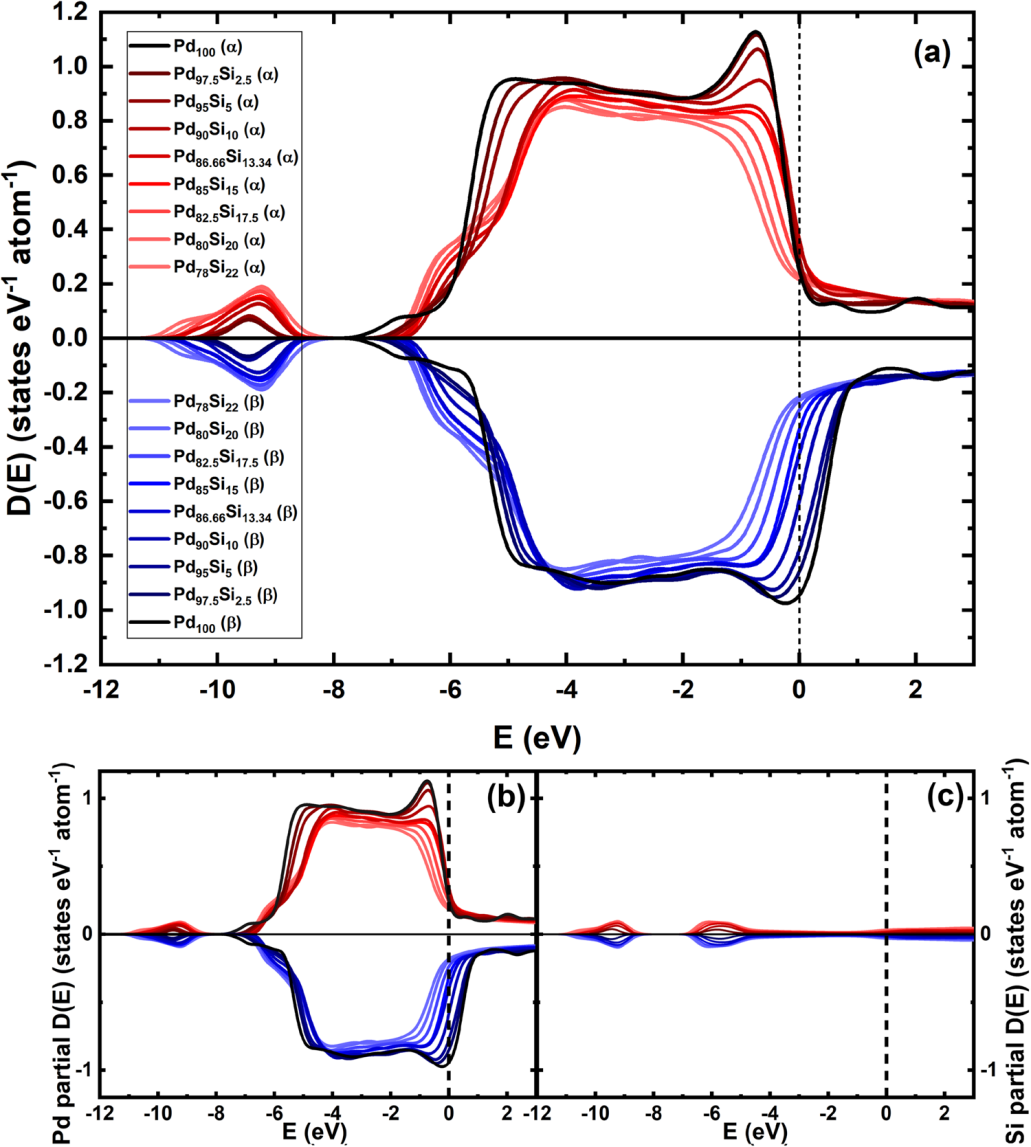
Spin up ($\alpha$ spins) and spin down ($\beta$ spins) densities of states for the 9 supercells. (**a**) Total densities of states. (**b**) Palladium partial densities of states. (**c**) Silicon partial densities of states. The ***net*** magnetic moment tends to zero as the silicon concentration increases.

**Figure 6:**
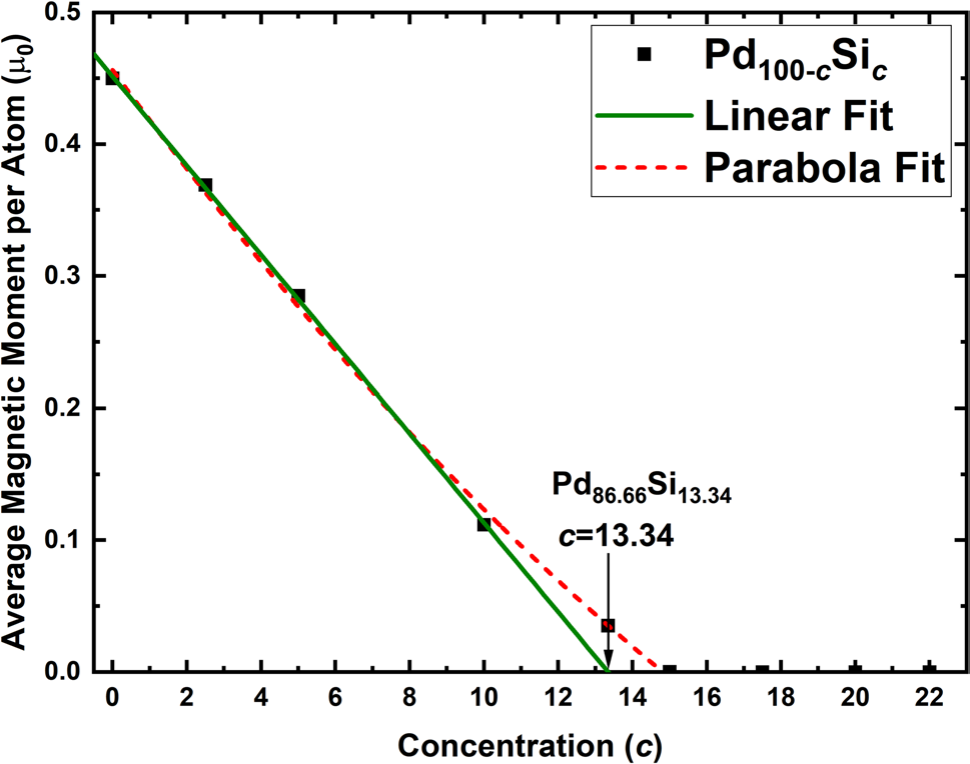
Average magnetic moment, AMM, per atom after the GO runs. The alloy *a*-Pd$_{86.66}$Si$_{13.34}$ was studied to investigate a possible linear fit to our magnetic results. The fit is not linear, green line, it is quadratic, red broken line. See text.

After the GO runs the Average Magnetic Moment, AMM, per atom was calculated to investigate the possible magnetism of the PdSi system. To do so $\alpha$ spins and $\beta$ spins densities of states were obtained for the 9 supercells and so were the areas under each curve for all the structures, and the differences in the areas were obtained; this gives the ***net*** magnetic moment for the supercell. To get the normalized results the net magnetic moment is divided by the number of atoms, the same for each cell. Figure 5 depicts all the $\alpha$ spin and $\beta$ spin results, and it can be observed that the asymmetry diminishes (the ***net*** AMM per atom tends to zero) as the concentration of silicon increases; see also Fig. 6. This indicates that increasing the concentration of silicon balances the loose spins in pure amorphous Pd up to about 15 at.%, giving a quantitative idea of the “defects” present in the amorphous pure structure. The symmetry of the partial silicon contributions to the up and down densities of states leads us to conclude that there is no ***net*** AMM in the silicon atoms. Moreover, the vanishing asymmetries of the $\alpha$ spins and $\beta$ spins contributions of the Pd atoms as *c* increases, indicates that the magnetism is associated to these atoms. More detailed studies are needed to inquire into these conclusions, and to discern the origin and evolution of magnetism.

It is difficult not to speculate about the differences between the results reported herein, and those of Müller et al.^16^ for the liquid counterpart, in the light of Sänger’s explanation^17^ of the experimentally determined total magnetic susceptibility, $\chi (T,c)$. Sänger’s decomposition indicates contributions from the *d*-spin paramagnetism, $\chi _{d} (T,c)$, the *s*/*p* band spin paramagnetism, $\chi _{s} (c)$, the Pd *d*-band orbital paramagnetism, $\chi _{orb} (c)$, and the total diamagnetism, $\chi _{dia} (c)$, of the constituents:

$$\begin{aligned} \chi (T,c) = \chi _{d} (T,c) + \dfrac{2}{3} \chi _{s} (c) + \chi _{orb} (c) + \chi _{dia} (c) \end{aligned}$$

**Figure 7:**
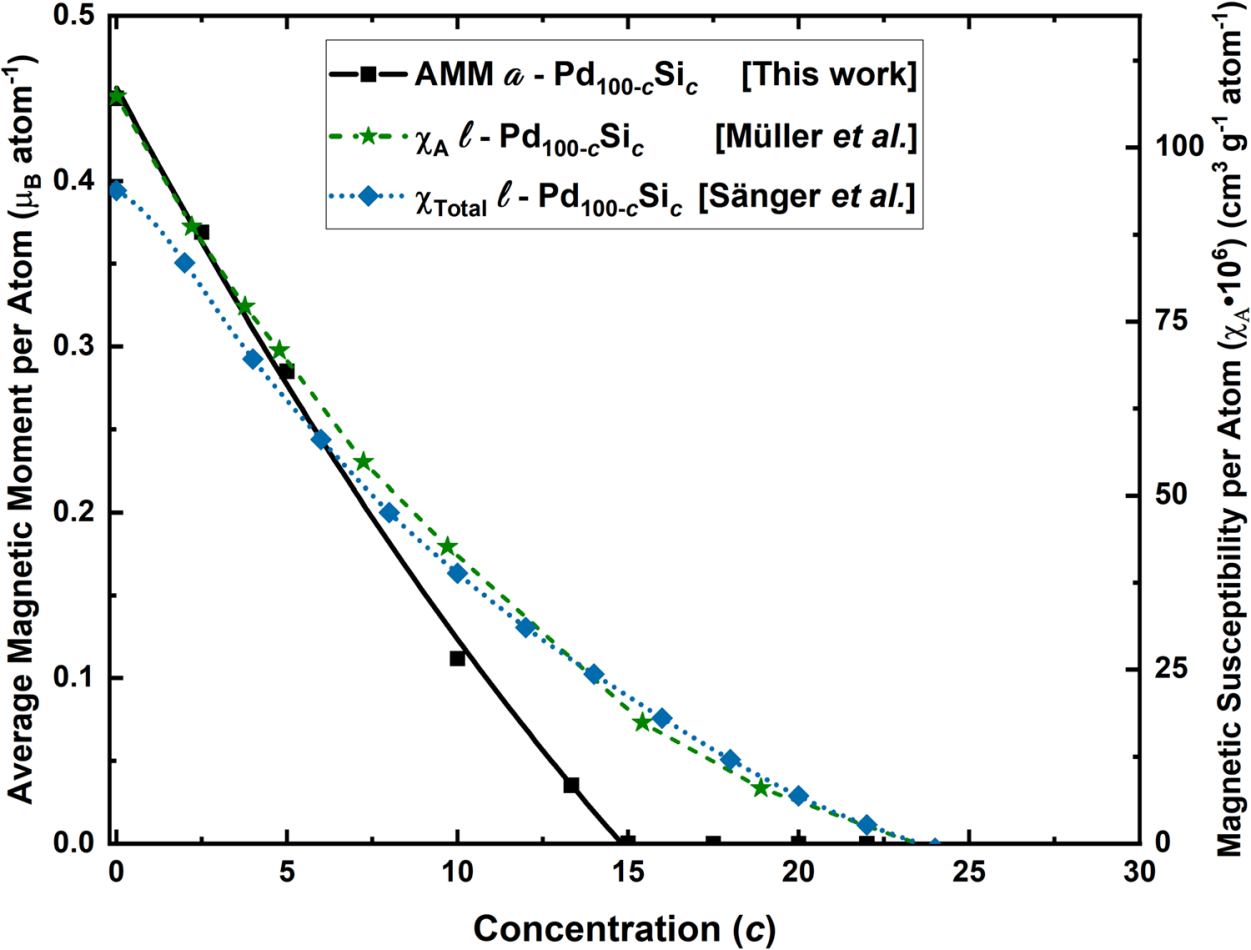
*Qualitative* comparison of our results for the AMM per atom of the solid *a*-PdSi alloys (vertical scale on the left) with the magnetic susceptibility measurements for the liquid counterpart (vertical scale on the right).

This expression indicates that the only temperature-dependent contribution to the magnetic susceptibility of the alloys appears in the *d*-contribution due to palladium, but no phase-dependent contributions are invoked. If the experimental susceptibility results (Figure 1 from Ref.^17^) are displaced downwards rigidly until the green curve in Fig. 7 and the simulational results coincide at 15 at.%, one would have to conclude that the terms that Sänger invoke are relevant for low concentrations in the liquid and are not as relevant for the amorphous. In fact, based on the results of the magnetism found for the amorphous pure, solid, palladium reported elsewhere^11^ the *d*-contribution seems to be more relevant and may account for higher values of magnetism in the amorphous solid PdSi alloys. However, more work is needed to elucidate the relevance of each contribution in the solid amorphous phases, and the why.

These naïve conclusions should be handled with care. To our knowledge, to date no semiphenomenological calculations, like Sänger’s, have been reported for solid amorphous PdSi alloys. It would be necessary to do a more rigorous estimation of any of the terms considered for the liquid alloys, that may be relevant for the solid ones, to reach reasonable conclusions.

It is important to mention that the possible magnetic cluster-like structures^8^ in the samples were not studied; these structures may give us an indication of the presence of nano-magnetism due to the presence of nano-domains. The structures have been mentioned in several places and should be interesting to study the feasibility of such constructions for small supercells like the ones reported here.

Our run for *c* = 13.34 (*a*-Pd$_{86.66}$Si$_{13.34}$) was excluded at first since no need to do it was anticipated, but when some of the curves were adjusted to the initial results the possible linear fit to the AMM as a function of Si concentration was considered as the best choice. However, When the run for this concentration was carried out the zero intercept of the line did not occur for *c* = 13.34 (the AMM per atom was non-zero for this value), then a parabolic fit was chosen to describe our results. The subsequent concentrations were as close to zero as one can expect and in Table 2 the magnetic moments found for the 9 samples are listed. The parabola fit is done for the pure and for the five lowest concentrations of silicon; the linear fit is done for the pure and for the silicon three lowest concentrations. The four highest concentrations (*c* = 15, 17.5, 20 and 22) have an AMM essentially null, see Fig. 6 and Table 2. Figure 7 displays a qualitative comparison of our results and the experimental liquid results of Müller et al.^16^.

**Table 2:** AMM per atom for the pure bulk, amorphous, sample of palladium and the eight alloys studied in this work.

Alloy	AMM $\left( \mu _{0} \right)$
Pd$_{78}$Si$_{22}$	$6.30 \times 10^{-7}$
Pd$_{80}$Si$_{20}$	$8.28 \times 10^{-7}$
Pd$_{82.5}$Si$_{17.5}$	$5.05 \times 10^{-7}$
Pd$_{85}$Si$_{15}$	$1.06 \times 10^{-4}$
Pd$_{86.66}$Si$_{13.34}$	0.04
Pd$_{90}$Si$_{10}$	0.11
Pd$_{95}$Si$_{5}$	0.29
Pd$_{97.5}$Si$_{2.5}$	0.37
Pd$_{100}$	0.45

Two curve fittings are shown in Fig. 6.

Byproducts of this investigation are the results for the positions of the maxima of the first prominent peaks of the PDFs, and the coordination numbers of Si around Pd and Pd around Pd; all these are compared with some experimental results, past and recent, in what follows. An analysis of the prominent peaks of the pPDFs is presented in Table 3 where the positions of the simulated peaks for first-neighbors Pd-Si and Pd-Pd are given so the comparison with experiment can be carried out. The experimental results give 2.40 Å obtained with neutron techniques (Table 4.3, p. 71 of Ref.^19^) for the first peak of the Pd-Si pPDFs and our findings indicate that the average of our 8 concentrations is 2.41 Å, a good agreement (Table 3). Nevertheless, an increasing subtle tendency is observed for the value of the positions of the maxima of these peaks when the concentration of Si increases. On the other hand, experimentally “the coordination of silicon by palladium in Pd-Si glasses varies from 6 to 7 with decreasing silicon content and extrapolates to 9 for pure (hypothetical) amorphous palladium”^19^. Our simulations show that the coordination of Si by Pd (Z$_{\text {Si-Pd}}$) systematically increases from 8.4 for Pd$_{78}$Si$_{22}$ to 9.2 for Pd$_{97.5}$Si$_{2.5}$ when the concentration of Si decreases, which agrees with the tendency of some recent experimental results^28,32^, as well as with the hard sphere model of Boudreaux^33^, and with the Pd$_{80}$Si$_{20}$ ab-initio value of Durandurdu^29^. For the palladium surrounded by Pd (Z$_{\text {Pd-Pd}}$) the coordination increases from 8.75 for Pd$_{78}$Si$_{22}$ to 11.07 for pure palladium, in contrast with the hard sphere model of Boudreaux that stays constant at around 10.5, for concentrations $10 \le c \le 30$^33^. Compare with the extrapolated experimental value of 9 quoted in Ref.^19^. See Table 4.

**Table 3:** Positions in Å for the first two prominent peaks (R$_{1-1}$ and R$_{1-2}$) of the pPDFs.

Alloy	$R_{1-1}$ (Å) Pd-Si	R$_{1-2}$ (Å) Pd-Pd
Pd$_{78}$Si$_{22}$	2.425	2.765
Pd$_{80}$Si$_{20}$	2.425	2.765
Pd$_{82.5}$Si$_{17.5}$	2.415	2.755
Pd$_{85}$Si$_{15}$	2.415	2.735
Pd$_{86.66}$Si$_{13.34}$	2.405	2.745
Pd$_{90}$Si$_{10}$	2.405	2.725
Pd$_{95}$Si$_{5}$	2.385	2.695
Pd$_{97.5}$Si$_{2.5}$	2.395	2.685
Pd$_{100}$	–	2.685
Average	2.409	2.728

The position R$_{1-1}$ of the simulated first-neighbor is, on average, 2.41 Å; the experimental value is 2.4 Å^19^.

**Table 4:** Some coordination numbers (Z) for PdSi alloys, experimental^19,28,32^ and simulational^32,33^, compared to our results.

Alloy	[This work]			[Boudreaux]			(exp.)[Ohkubo]			(exp.)[Suzuki]			[Durandurdu]		
	Z$_{\text {Pd-Si}}$	Z$_{\text {Si-Pd}}$	Z$_{\text {Pd-Pd}}$	Z$_{\text {Pd-Si}}$	Z$_{\text {Si-Pd}}$	Z$_{\text {Pd-Pd}}$	Z$_{\text {Pd-Si}}$	Z$_{\text {Si-Pd}}$	Z$_{\text {Pd-Pd}}$	Z$_{\text {Pd-Si}}$	Z$_{\text {Si-Pd}}$	Z$_{\text {Pd-Pd}}$	Z$_{\text {Pd-Si}}$	Z$_{\text {Si-Pd}}$	Z$_{\text {Pd-Pd}}$
Pd$_{70}$Si$_{30}$	–	–	–	3.29	8.40	10.34	–	–	–	–	–	–	–	–	–
Pd$_{78}$Si$_{22}$	2.40	8.40	8.75	2.19	8.26	10.72	–	–	–	–	–	–	–	–	–
Pd$_{80}$Si$_{20}$	2.18	8.19	9.28	2.05	8.44	10.22	-	-	-	1.64	6.56	10.60	2.17	8.70	10.77
Pd$_{82}$Si$_{18}$	–	–	–	1.78	8.36	10.49	1.80	8.00	10.60	–	–	–	–	–	–
Pd$_{82.5}$Si$_{17.5}$	1.88	8.82	9.53	–	–	–	–	–	–	1.38	7.58	10.60	–	–	–
Pd$_{85}$Si$_{15}$	1.54	8.85	9.70	1.44	8.46	10.59	–	–	–	–	–	–	–	–	–
Pd$_{86.66}$Si$_{13.34}$	1.34	8.88	9.90	–	–	–	–	–	–	–	–	–	–	–	–
Pd$_{90}$Si$_{10}$	1.01	8.91	10.29	0.85	8.21	10.65	–	–	–	–	–	–	–	–	–
Pd$_{95}$Si$_{5}$	0.49	9.00	10.48	–	–	–	–	–	–	–	–	–	–	–	–
Pd$_{97.5}$Si$_{2.5}$	0.26	9.17	10.76	–	–	–	–	–	–	–	–	–	–	–	–
Pd$_{100}$	–	–	11.07	–	–	–	–	–	–	–	–	–	–	–	–

## Conclusions

After having found magnetic properties in amorphous bulk palladium^11^, possible manifestations of magnetism in systems based on Pd, that would give certainty to our findings in the amorphous and in the amorphous/nano-porous phases, were considered. Hence the contamination of amorphous Pd with the isotopes H, D and T, *a*-Pd (H/D/T)$_{x}$ (where *x* is the ratio of the contaminants) were studied, and found that, for ratios $x < 1$, *a*-Pd(H/D/T)$_{x}$ is magnetic^12^. Then the next step was to study amorphous Pd$_{100-c}$Si$_{c}$ for *c* less than or equal to 22; the results are reported herein.

It is clear that the amorphicity in Pd is responsible for the magnetism in all these materials and a more systematic study, both experimental and computational, should reveal new materials, based on palladium, that are magnetic. Also, a more detailed study is needed to clarify the nature of these magnetic properties and to enquire into the possible existence of spin-glass domains, or spin-glass clusters at the nano level. This is underway.

Since the liquid Pd-Si alloys display magnetism, and since the structural characteristics of liquids and amorphous metallic alloys are somewhat similar, it was expected that the solid, glassy metals, *a*-Pd$_{100-c}$Si$_{c}$ should display magnetism, and they do, as shown in this paper. Other studies of Pd-based alloys are in order to investigate how wide-spread these phenomena are and to identify the type of magnetic ordering that occurs in these alloys (See chapter 20 of Ref.^8^, for an analysis of the variety of magnetic phenomena; in particular, magnetism in glass clusters).

A collateral inference of our studies is the otherwise evident conclusion that ab initio studies better describe the topological aspects (position of the nearest peaks) of the structure and better describe the quantum mechanical nature of the chemical bonding.
